# First human case of catheter-related blood stream infection caused by *Staphylococcus schleiferi* subspecies *coagulans*: a case report and literature review

**DOI:** 10.1186/s12941-021-00474-3

**Published:** 2021-09-25

**Authors:** Tatsuya Kobayashi, Mahoko Ikeda, Yuki Ohama, Koji Murono, Kazuhiko Ikeuchi, Satoshi Kitaura, Koh Okamoto, Shu Okugawa, Soichiro Ishihara, Kyoji Moriya

**Affiliations:** 1 Department of Infectious Diseases, University of Tokyo Hospital, Tokyo, Japan; 2 Department of Surgical Oncology, The University of Tokyo Graduate School of Medicine, Tokyo, Japan; 3 Department of Infection Control and Prevention, University of Tokyo Hospital, 7-3-1, Hongo, Bunkyo-ku, Tokyo, 113-8655 Japan

**Keywords:** *Staphylococcus schleiferi* subspecies *coagulans*, Catheter-related bloodstream infection, Matrix-assisted laser desorption/ionization time-of-flight mass spectrometry, 16S ribosomal RNA sequencing

## Abstract

**Background:**

*Staphylococcus schleiferi* is a gram-positive pathogenic coccus which causes canine skin and ear infections. Only four cases of human infection caused by *Staphylococcus schleiferi* subspecies *coagulans* have been reported. Herein, we present the first case of catheter-related bloodstream infection caused by *S. schleiferi* subspecies *coagulans*.

**Case presentation:**

A 62-year-old Japanese man was admitted to our hospital for examination of sigmoid colon tumor. During hospitalization, he had fever, shaking chills, and swelling at the peripheral venous catheter insertion site. Two sets of blood cultures were positive for *S. schleiferi* subspecies *coagulans* which was confirmed using matrix-assisted laser desorption/ionization time-of-flight mass spectrometry (MALDI-TOF MS), 16S ribosomal RNA sequencing and the coagulase test. The patient was successfully treated without relapse.

**Conclusion:**

To our knowledge, this is the first report of catheter-related bloodstream infection caused by *S. schleiferi* subspecies *coagulans. S. schleiferi* subsp*. coagulans* can be pathogenic in humans, and MALDI-TOF MS can contribute to accurate identification of *S. schleiferi* subspecies *coagulans.*

## Background

*Staphylococcus schleiferi* is associated with otitis externa and pyoderma in dogs [1]. It has two subspecies, *S. schleiferi* subsp*. schleiferi* and *S. schleiferi* subsp*. coagulans* that are coagulase-negative and coagulase-positive, respectively. There are multiple case reports of *S. schleiferi* infection including skin and soft tissue infections, wound and surgical site infections [2], device infections [3], urinary tract infection [4], and endocarditis [5]. A review of 28 case series of *S. schleiferi* infection has been reported in 2001 [6], and all isolates were coagulase-negative.

However, only four cases of human infection caused by *S. schleiferi* subsp*. coagulans* have been reported. Here, we report the first case of catheter-related bloodstream infection caused by *S. schleiferi* subsp*. coagulans*.

## Case presentation

A 62-year-old Japanese man presented to a local gastroenterology clinic with narrow stools. He had hypertension and prostatic hypertrophy. His only regular medication was amlodipine. Colonoscopy revealed a sub-circumferential tumor in the sigmoid colon, and the colonoscope did not pass through the tumor. He was referred to our hospital for further examination of a sigmoid colon tumor. Bowel obstruction due to sigmoid colon cancer was suspected, and he was admitted to our hospital. Fasting and fluid administration were performed until surgical resection of the tumor.

Seven days after admission, he had a fever of 38.1 °C with shaking chills but was alert and oriented and not in acute distress. Additionally, his vital signs were as follows: pulse rate 72 beats/min, blood pressure 118/60 mmHg, respiratory rate 16 breaths/min, and oxygen saturation 98% while breathing ambient air. Physical examination revealed redness, swelling, and tenderness at the short peripheral venous catheter insertion site in his right forearm. He did not have a heart murmur, neurological abnormalities, or other abnormalities on physical examination. He denied a history of trauma, intravenous drug use, and contact with animals including dogs.

Laboratory tests showed the following results: white blood cell count 9000/µl (neutrophil 79.9%) and C-reactive protein 5.72 mg/dl. His serum creatinine and liver function tests were normal. A computed tomography scan showed no abnormal findings except for the previously known sigmoid colon tumor.

Because catheter-related bloodstream infection was suspected, a peripheral venous catheter was replaced. Piperacillin/tazobactam (4.5 g every 8 h) was initiated after two sets of blood cultures were collected. The blood cultures were collected from separate venipuncture sites by using aseptic non-touch technique. On the following day, both sets of blood cultures were positive for clusters of gram-positive cocci. Therefore, vancomycin (1 g every 12 h) was initiated additionally.

The isolate was identified using matrix-assisted laser desorption/ionization time-of-flight mass spectrometry (MALDI-TOF MS), using a MALDI Biotyper (Bruker Daltonics, Bremen, Germany, Version 4.0) as *S. schleiferi* subsp*. coagulans* (log score 1.945).

The results were confirmed using 16S ribosomal RNA sequencing. Comparison with sequences in the BLAST database (http://www.ncbi.nlm.nih.gov/BLAST) revealed that the sequence was 100% identical to that of *S. schleiferi* (GenBank accession number AP014944.1). The isolate was free coagulase-positive and thus, was identified as *S. schleiferi* subsp*. coagulans*. Coagulase activity was tested by using rabbit plasma (Eiken Chemical Co., Ltd., Tokyo, Japan).

Antimicrobial susceptibility testing was performed using the MicroScan WalkAway system (Beckman Coulter, Tokyo, Japan). The isolate was susceptible to all antibiotics tested including oxacillin, cefazolin, clindamycin, and vancomycin according to the Clinical and Laboratory Standards Institute M100-S29 [7]. The minimum inhibitory concentration for oxacillin was ≤ 0.25 μg/ml.

The isolate did not produce Staphylococcal enterotoxin (SE, SEA to SED) or toxic shock syndrome toxin-1 (TSST-1) as per reversed passive latex agglutination assay (Denka Co., Ltd., Tokyo, Japan).

The patient’s antibiotic treatment was switched to cefazolin (1 g every 8 h) on day 5 based on the results of the antimicrobial susceptibility testing. His fever and chills improved following antibiotic treatment. Repeated blood cultures after antibiotic treatment were negative. He was treated for eight days with intravenous antibiotic therapy. The patient successfully underwent laparoscopic sigmoid colectomy without relapse of *S. schleiferi* bacteremia.

## Discussion and conclusion

*S. schleiferi* was first reported as a new species in 1988 [8], and *S. schleiferi* subsp*. coagulans* can be differentiated from *S schleiferi* subsp*. schleiferi* based on coagulase production [9]. *S. schleiferi* is frequently isolated from the skin and external ear of dogs [10] and is recognized as one of the pathogens causing canine otitis externa [9].

While various human cases of *S. schleiferi* infection have been described previously, human infection caused by *S. schleiferi* subsp*. coagulans* is rare. Only four cases of *S. schleiferi* subsp*. coagulans* infection have been reported previously including two cases of endocarditis [5, 11], a case with left ventricular assist device infection [12], and a case with bacteremia (entry site undescribed) [13] (Table 1). None of the patients died of *S. schleiferi* subsp*. coagulans* infection.

**Table 1:** Characteristics of cases of human infection caused by *Staphylococcus schleiferi* subspecies *coagulans*

Infection focus	Age/sex	Definitive antibiotic treatment	Methicillin susceptibility	Outcome	References
Endocarditis	78/M	Benzylpenicillin, rifampin, gentamicin	Susceptible	Survived	Leung et al. [5]
Endocarditis	58/M	Vancomycin, rifampin, gentamicin	Resistant	Successfully treated (died of liver failure)	Kumar et al. [12]
Left ventricular assist device infection	55/F	Dicloxacillin, cephalexin	Susceptible	Survived	Thibodeau et al. [13]
Bacteremia (entry site undescribed)	66/M	Vancomycin	Resistant	Survived	Swe et al. [14]
Catheter related bloodstream infection	62/M	Cefazolin, clindamycin	Susceptible	Survived	This report

*M* male, *F* female

Both sets of blood cultures taken from the patient were positive for *S. schleiferi* subsp*. coagulans* and there was redness, swelling, and tenderness at the peripheral venous catheter insertion site. These clinical symptoms were resolved after withdrawal of the peripheral venous catheter. There was no other alternative explanation for bacteremia, and *S. schleiferi* subsp*. coagulans* can colonize on skin of human, especially who has contact with dogs [14]. For these reasons, we diagnosed his condition as catheter-related bloodstream infection. However, the patient denied contact with the dogs before admission. In addition, the onset of infection was a week after admission. Therefore, it is unclear how *S. schleiferi* subsp*. coagulans* colonized the patient’s skin in this case.

There are multiple reports that coagulase-negative staphylococci can produce SE alone or in combination with TSST-1 [15–17]. However, the *S. schleiferi* subsp*. coagulans* strain did not produce SEA to SED or TSST-1 in our case. Although the possession of SE genes was reported in 86% (18/21 strains) of coagulase-positive *S. schleiferi* detected from dogs, the production of SEA to SED and TSST-1 has not yet been reported [18]. As *S. schleiferi* subsp*. coagulans* infection is rare in humans and there are a limited number of case reports, further studies are needed to clarify the frequency of SE/TSST-1 production in *S. schleiferi* subsp. *coagulans* infection.

Various laboratory tests were performed to identify *S. schleiferi* subsp*. coagulans* in previous reports. Two reports used Vitek system (bioMérieux Vitek Inc., Hazelwood, Missouri) [5, 12]. One report used 16S ribosomal DNA sequencing, nested PCR, and the MicroScan WalkAway system (Dade-Behring, West Sacramento, California) [5, 11, 12]. Our case is the first to use MALDI-TOF MS, 16S ribosomal RNA gene sequencing for identification of *S. schleiferi* subsp*. coagulans*. *S. schleiferi* subsp*. coagulans* could have been mistaken for *Staphylococcus aureus* because both are free coagulase positive [5, 19]. Accurate identification of *S. schleiferi* subsp*. coagulans* can be expected by combining MALDI-TOF MS and biochemical properties test.

Non-aureus *Staphylococci*, especially coagulase-negative *Staphylococci* are often resistant to methicillin [20], however, two previous cases and the present case were susceptible to methicillin [5, 11–13].

In summary, we present the first case of catheter-related bloodstream infection caused by *S. schleiferi* subsp*. coagulans*. The organism was identified using MALDI-TOF MS, 16S ribosomal RNA gene-sequencing analysis, and free coagulase test. Therefore, although human infection is rare, *S. schleiferi* subsp*. coagulans* can be pathogenic in humans.

## Data Availability

All data analyzed in this study are included in this published article. Related information is accessible under request to the corresponding author.

## Abbreviations

*S. schleiferi*: *Staphylococcus schleiferi*
*S. schleiferi * subsp*. schleiferi*: *Staphylococcus schleiferi* subspecies *schleiferi*
*S. schleiferi* subsp*. coagulans*: *Staphylococcus schleiferi* subspecies *coagulans*
MALDI-TOF MS: Matrix-assisted laser desorption/ionization time-of-flight mass spectrometry
SE: Staphylococcal enterotoxin
TSST-1: Toxic shock syndrome toxin-1
